# Potential of Cellulose Functionalized with Carboxylic Acid as Biosorbent for the Removal of Cationic Dyes in Aqueous Solution

**DOI:** 10.3390/molecules23040743

**Published:** 2018-03-23

**Authors:** Lucinaldo dos Santos Silva, Jhonatam de Oliveira Carvalho, Roosevelt Delano de Sousa Bezerra, Mateus Soares da Silva, Francisco José Lustosa Ferreira, Josy Anteveli Osajima, Edson Cavalcanti da Silva Filho

**Affiliations:** 1 Açailândia Campus, Federal Institute of Maranhão, IFMA, Açailândia, MA 65930-000, Brazil; ssdlucinaldo@gmail.com (L.d.S.S.); jhonatam.carvalho@ifma.edu.br (J.d.O.C.); 2 Teresina-Central Campus, Federal Institute of Piauí, IFPI, Teresina, PI 64000-040, Brazil; rooseveltdsb@ifpi.edu.br; 3 Interdisciplinary Laboratory for Advanced Materials—LIMAV, UFPI, Teresina, PI 64049-550, Brazil; mateusufpi@gmail.com (M.S.d.S.); franciscojufpi@gmail.com (F.J.L.F.); josy_osajima@yahoo.com.br (J.A.O.)

**Keywords:** cellulose, modification, description, adsorption, cationic dye

## Abstract

In the last decade, adsorption has been used to minimize the pollution caused by dyes, which represents a serious environmental problem. In this context, this work reports the preparation of phthalic anhydride-modified cellulose (PhCel), through the reaction of cellulose (Cel) with phthalic anhydride (Ph). The efficiency of the reaction was observed by elemental analysis, Fourier Transform Infrared (FTIR) spectroscopy, X-ray diffraction (XRD) and thermogravimetry/derivative thermogravimetry (TG/DTG). The adsorbent matrix (Cel and PhCel) was used in the removal of crystal violet (CV) and methylene blue (MB) dyes in aqueous medium. In the kinetic study, the experimental data obtained had the best fit to the pseudo-first-order model. In general, the isotherms obtained at different temperatures had a best fit to the model proposed by Langmuir, and the CV and MB adsorption process in adsorbent matrixes can be favored strictly by hydrogen bonds and/or electrostatic interactions for Cel and electrostatic interactions for PhCel.

## 1. Introduction

In the last decade, studies have been carried out to minimize the pollution caused by industrial effluents, since the legislation is becoming increasingly rigid in relation to the limits of effluent emission into the environment. Among the effluents, anionic [1,2,3] and cationic [4,5,6,7] dyes, which represent a serious environmental problem, can be highlighted, since in many cases these substances show great resistance to chemical, photochemical or biological degradation methods [8]. In addition, pollution from this class of compound damages several biochemical processes in living beings, such as photosynthesis in aquatic environments, that are of fundamental importance for the maintenance of life on Earth [9].

Cationic dyes are widely employed in the dyeing process of leathers, paper and acrylic fibers; this process involves, as a final step, washing in flowing water to remove excess original dye or hydrolyzed dye not attached to the fibers in the previous steps due to low fixing rates. This process discards 10–15% of the dyes as effluent, polluting the environment and affecting aquatic organisms [3].

Violet crystal (CV) and methylene blue (MB), when dumped into the environment, can affect people in a variety of ways, such as when washing, bathing or drinking. These are not biodegradable substances and can cause various diseases and disorders in living organisms, even at low concentrations [10,11]. In this context, numerous dye removal techniques have attracted attention from chemists. Many of them are still in the experimental phase, such as precipitation, ion exchange, adsorption, filtration and electrodeposition [12]. Among all the techniques mentioned, adsorption is an important method for removal of dyes in aqueous media [2,13].

Currently, several adsorbents have been used in aqueous dye adsorption processes [14], especially cellulose, which, being the most abundant polysaccharide in Nature, arouses great attention mainly due to the reactivity of its hydroxyl groups [15,16,17]. Its ability to adsorb dyes can be increased by suitable chemical modification of its surface in order to incorporate organic molecules containing basic groups, mainly those rich in nitrogen, sulfur and oxygen. Almost always this modification involves non-complicated chemical processes, to give a new surface which generates, for example, an ionic and or covalent interaction capacity with dyes in aqueous medium [18,19]. 

One of the processes studied for the modification of cellulose is based on the synthesis to incorporate in the polymer structure derived from anhydrides, where maleic [6], succinic [17] and phthalic [20] anhydride can be highlighted. This synthesis is important because it can immobilize on the biopolymer carboxylic groups that can increase the adsorption capacity of cationic dyes in aqueous medium. Thus, this work describes the modification of the cellulose surface with phthalic anhydride in the absence of solvent. Cel and PhCel were characterized by elemental analysis, FTIR, XRD, TG/DTG, and determination of carboxylic groups, and applied to the removal of crystal violet and methylene blue dye where pH, time, concentration and temperature were evaluated. Finally, the experimental data were fitted to different physical-chemical isotherm models.

## 2. Results and Discussion

### 2.1. Characterization

**Figure 1:**
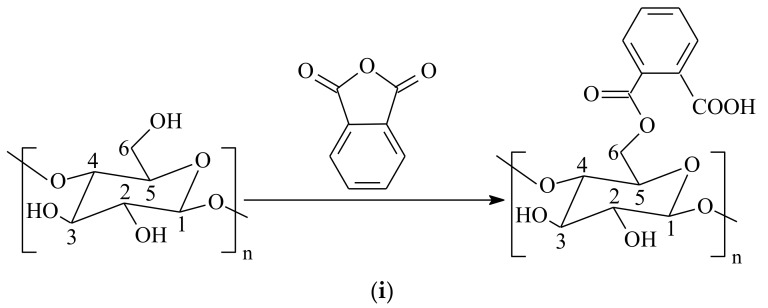

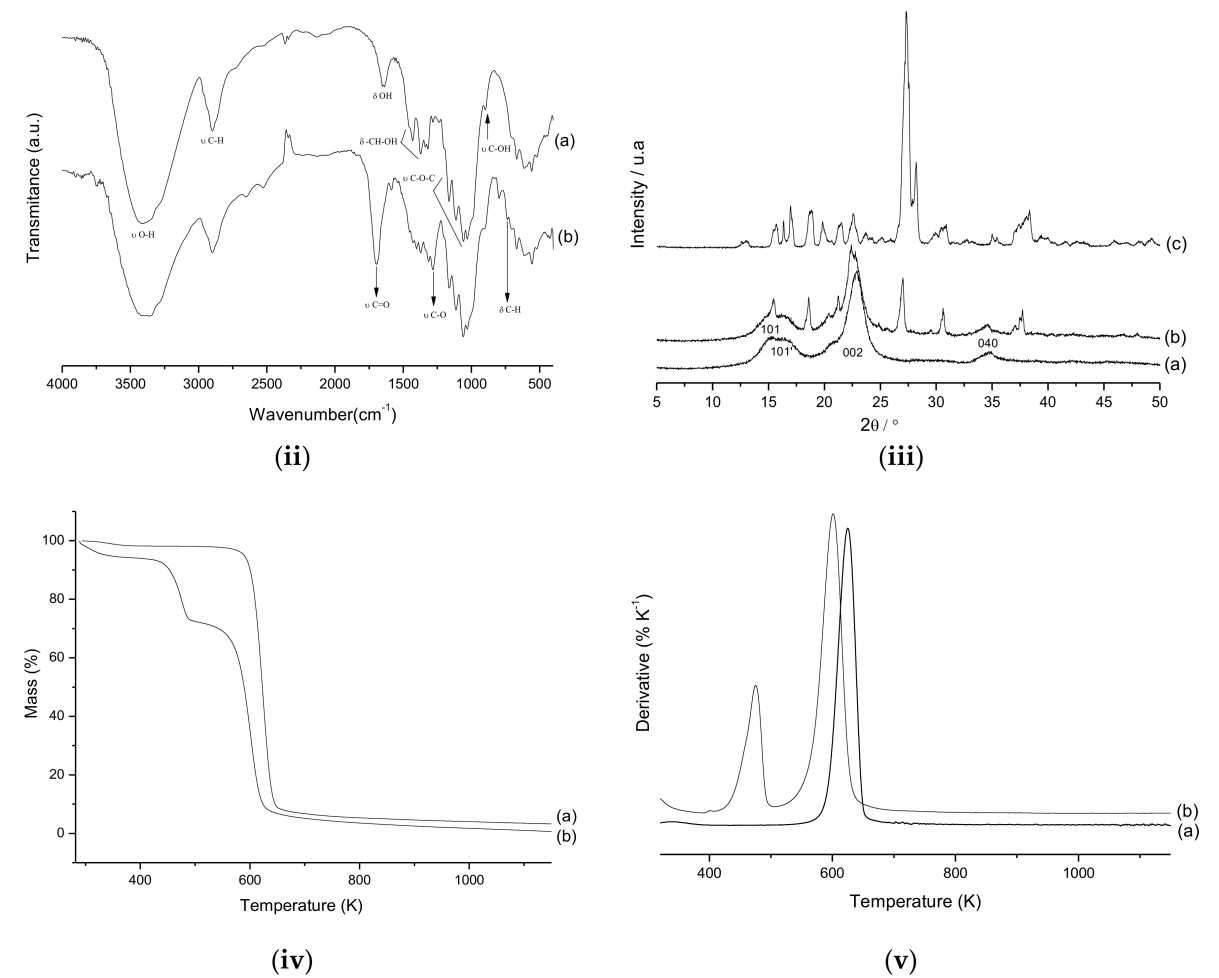
(**i**) Reaction scheme of the synthesis process; (**ii**) Fourier-transform infrared spectra of Cel (a) and PhCel (b); (**iii**) X-ray diffractograms of Cel (a), PhCel (b) and Ph (c); (**iv**) TG Curves of Cel (a) and PhCel (b); (**v**) DTG Curves of Cel (a) and PhCel (b).

After the reaction of the cellulose with phthalic anhydride, the materials (Cel and PhCel) were characterized by elemental analysis of carbon and hydrogen. From this analysis, 46.14 ± 0.05% C and 5.69 ± 0.08% H were obtained in PhCel, differing from the precursor polymer (41.47 ± 0.15% C and 6.27 ± 0.1% H). With this, we can observe that there was a significant increase in the percentage of carbon and a significant decrease in the percentage of hydrogen in the modified cellulose when compared to the pure cellulose. In addition, in the determination of carboxyl groups by reverse titration, 0.40 ± 0.03 and 3.56 ± 0.10 mmol·g^−1^ were determined for Cel and PhCel, respectively. These differences are related to the incorporation of carboxylic groups in PhCel according to the reaction scheme proposed in Figure 1i. From these results, we can observe that the amount of acid groups increased by nine times after the proposed modification.

The infrared spectra of Cel and PhCel are shown in Figure 1ii. The precursor biopolymer in Figure 1ii(a) shows the presence of OH groups due to the band appearing in the region between 3500 and 3200 cm^−1^, which are vibrations corresponding to the OH stretch of the polymer chain ν(CH–OH) and ν(CH_2_–OH). Another important vibration in the cellulose spectrum appearing in the 3000–2800 cm^−1^ region is attributed more precisely to ν(C–H) of methyl and methylene groups.

The band at 1639 cm^−1^ corresponds to the deformation of the primary and secondary δ-OH hydroxyl groups and the bands in the region 1500–1200 cm^−1^ correspond to the deformation of the primary and secondary δ(CH–OH) hydroxyl groups. The 1169, 1110 and 1058 cm^−1^ bands refer to the β-1,4-glycosidic ether stretch, ν(C–O–C) and those below 1000 cm^−1^ are attributed to the uptake of alcoholic groups of the polymer chain [2,21].

When comparing the pure cellulose (Cel) spectrum shown in Figure 1ii(a) to the PhCel spectrum shown in Figure 1ii(b), the bands at 2652 and 2500 cm^−1^ correspond to the carboxylic acid dimers as do those at 1691 cm^−1^ ν(acid C=O), and the appearance of a band at 1280 cm^−1^ is related to ν(C–O esters). The band at 745 cm^−1^ refers to δ(C–H) outside the *ortho*-benzene disubstituted ring of the anhydride group incorporated into the biopolymer structure. The changes of the bands at 1169, 1110 and 1058 cm^−1^ can be explained by a conformational change due to the presence of the modifying groups, which, because of their bulky nature, distort the uniform structure of the cellulose chains, affecting the β-1,4-glycosidic bond, but without breaking it. The absence of bands at 1800 and 1850 cm^−1^ confirms that the material obtained is free of unreacted phthalic anhydride. Based on the differences shown, especially the appearance of the carbonyl group and ν(ester C–O), we can clearly indicate the binding of the anhydride molecules to the polymer structure of the biopolymer [7,22].

In Figure 1iii, the X-ray diffractograms for Cel, PhCel and Ph are shown. The cellulose used has different peaks characteristic of microcrystalline cellulose: 2θ = 14.9° (101), 16.6° (101′), 22.8° (002) and 34.9° (040). Comparing the diffractograms, the displacement of the 2θ peak from 22.8° (Figure 1iii(a)) to 22.3° (Figure 1iii(b)) and the appearance of the peak 2θ = 18.5° (Figure 1iii(b)) can be observed. These changes indicate that the proposed reaction occurred as observed in a similar study [7]. It is important to highlight some peaks in the diffractogram of PhCel arising from the phthalic anhydride incorporated in the cellulose; these may be related to the C–O–C group attached to the aromatic ring and the polymer chain of the cellulose (see Figure 1i), or could be attributed to a new superstructure formed during the synthesis. The new phase may have been favored because of the presence of the aromatic ring in the modified biopolymer. The aromatic rings have π-stacking capacity, in which the rings are disposed parallel to one another forming an organized structure in the biopolymer. Indices of this new phase were observed in the infrared spectrum of this material, in which it was possible to observe changes in the bands referring to the β-1,4-glycosidic bonds. The changes are probably related due to the distortions of the aliphatic ether bond caused by the bulky modifying group, as well as by the tensions they provide in the biopolymer matrix [7,20]. The crystallinity index (*CrI*) proposed by the method of Segal et al. [23], was determined using Equation (1):

(1) $$CrI=\frac{\left(I_{002}-I_{am}\right)}{I_{002}}\times 100$$

where *I*_002_ is the intensity of the crystalline portion (22° < 2θ < 23°) and *I_am_* is the intensity of the amorphous portion (18° < 2θ < 19°) [6,23]. 

Thus, a *CrI* for pure cellulose of 74.99% was obtained, whereas for modified cellulose it was 58.64%. This reduction in the *CrI* of the cellulose after the chemical modification accords with the results discussed above, because the proposed reaction can favor the disorganization of the outer chains and probably a decrease in the fiber size, which causes a disturbance in the inter- and intramolecular bonds of hydrogen, generating a new organized structure, thus reducing the crystallinity of the biopolymer.

Figure 1iv shows the thermogravimetric (TG) curves for Cel and PhCel and, in Figure 1v, the derivative TG curves (DTG). We can observe the decomposition of the pure cellulose in a single event, in a temperature range between 536 and 687 K, corresponding to a total mass loss of 91.76% correlated to adsorbed physical water, condensation of hydroxyls on carbons 2 and 3, and decomposition of the organic structure of the polymer chain. In the biopolymer modified with phthalic anhydride, decomposition occurs in two events; the first event between 410 and 503 K occurs with a mass loss of 21.35% corresponding to the loss of incorporated functional groups, and the second between 515 and 672 K occurs with a loss of mass of 65.55%, related to the decomposition of the organic structure, as observed in most modified biopolymers. It is important to note that the PhCel material favors water adsorption when compared to Cel, due to the fact this material has carboxylic acid groups on its surface. Thus, it can be observed in Figure 1iv that the mass loss related to physsisorbed water for the Cel occurs between 298–414 K with a mass loss of 1.76%, while the PhCel loss occurs between 298–381 K with a mass loss of 4.07% [2,6]. The mass losses in PhCel can be observed through the two peaks observed in the DTG curve in Figure 1v. 

**Figure 2:**
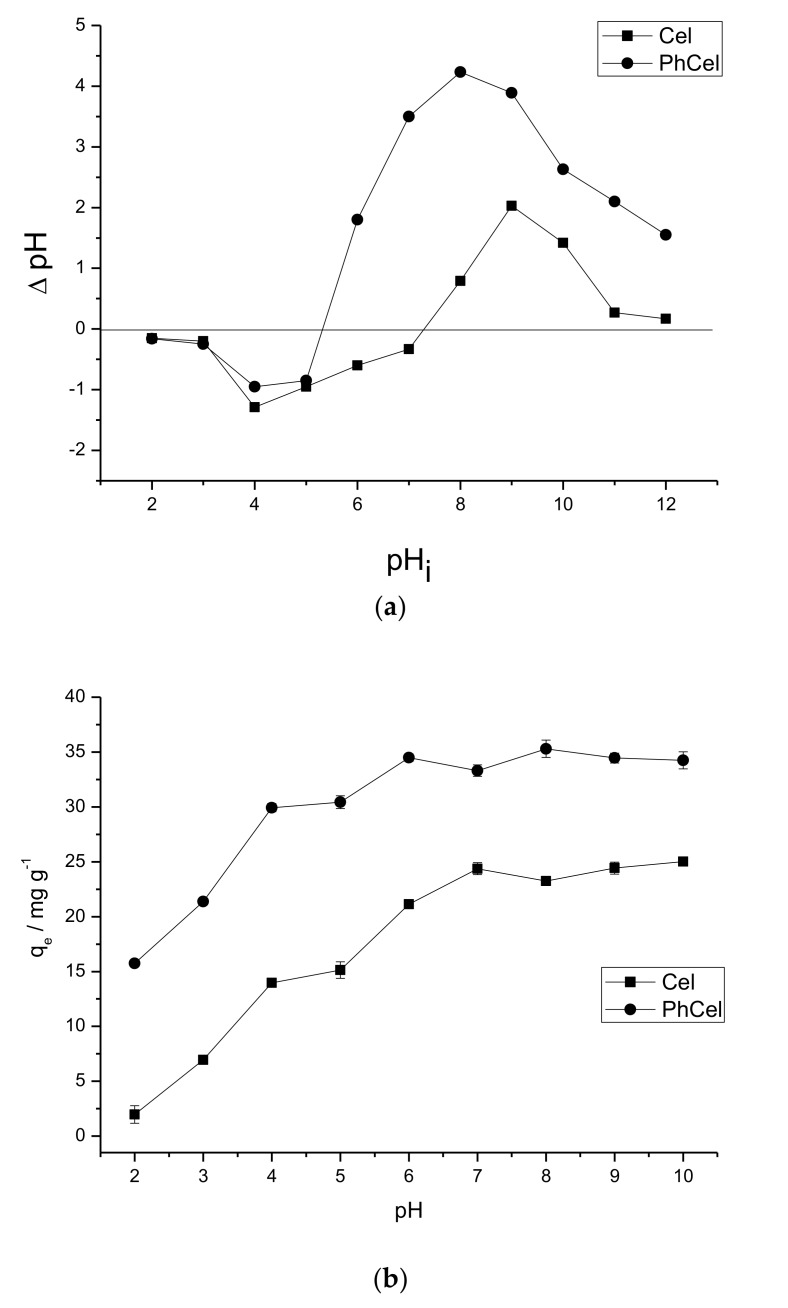

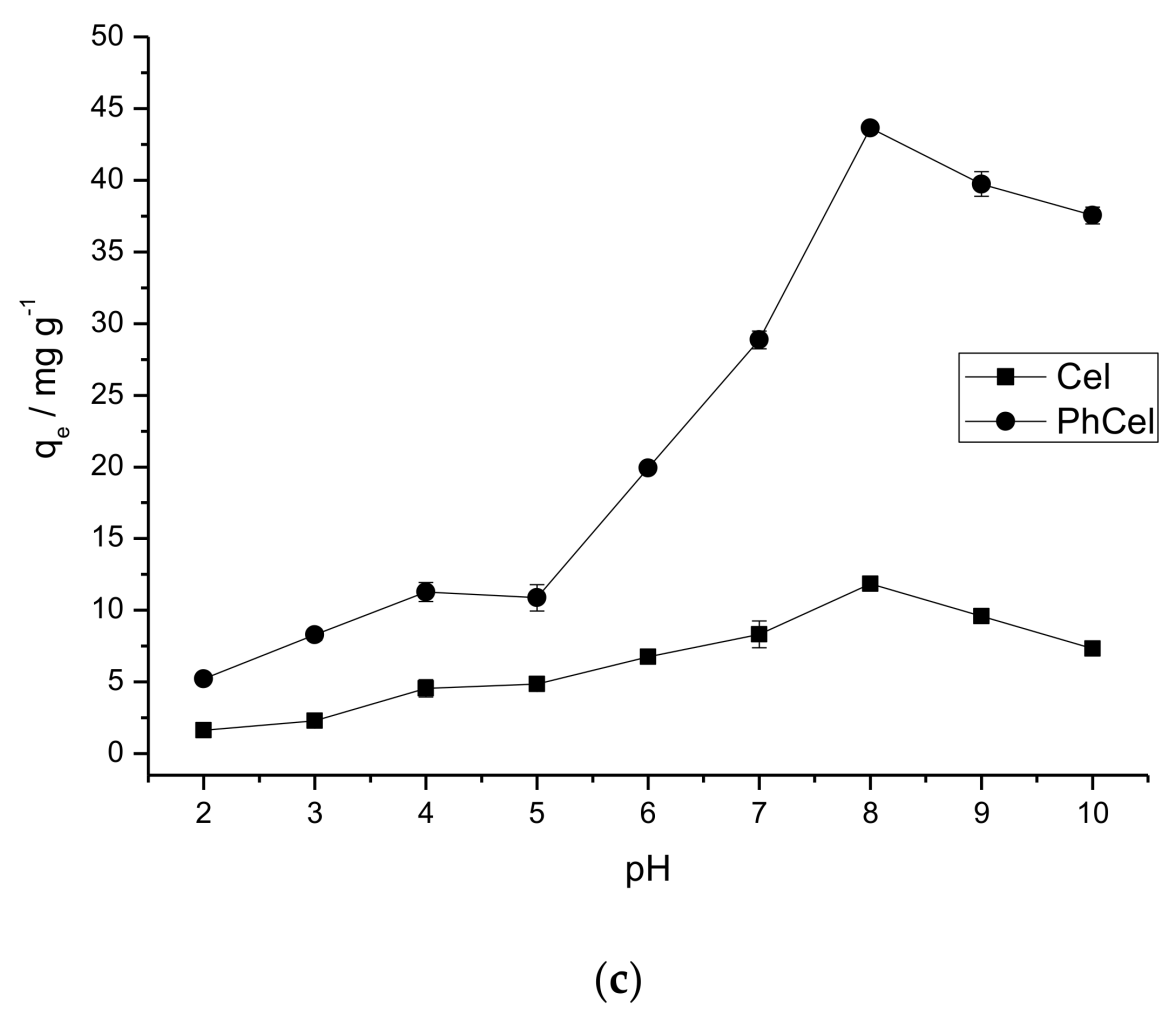
(**a**) Point of zero charge of Cel and PhCel. Effect of pH on the adsorption of (**b**) CV and (**c**) MB onto Cel or PhCel.

Figure 2a shows the graph of the zero charge potential of the adsorbent matrices, which shows the behavior of the surface charge of the Cel or PhCel. At pHi 2.0 to 4.0, a small retention of protons occurs in the materials, with a slight increase in the pH_f_ value. After pH_i_ 5, protons retention decreases gradually reaching pH_pzc_ (point of zero charge) at 7.30 and 5.30 for Cel and PhCel, respectively. Above pH_pzc_, the surface of the materials begins to release protons into the medium, and consequently the pH value decreases. This process occurs until around pH_i_ 9.0 for Cel and 8.0 for PhCel and, after this, the attraction of the protons by the surface of the materials begins again, increasing the pH of the medium [2,17]. The difference of the pH_pzc_ of the adsorbent matrices can be related to the incorporation of the phthalic anhydride molecule in the cellulose, which generates a surface with carboxylic groups that are easily deprotonated, corroborating to the balance between the charges (pH_pzc_) occurring at lower pH in PhCel when compared to Cel. These results confirm that the pH of the medium may influence the surface of the biopolymers, that is, the ions (H^+^ or OH^−^) present in solution may interact with the active sites of the biopolymers, thus altering the charge. 

### 2.2. Dye Adsorption

The study of the influence of pH on adsorption of crystal violet dye (Figure 2b) and methylene blue (Figure 2c) on the surface of Cel or PhCel, shows that for pure cellulose, the CV adsorption capacity remains practically constant from pH 7.0, and for PhCel, from pH 6.0, a fact observed in a similar study [6]. For adsorption of MB on pure and modified cellulose, the highest capacity was at pH 8.0, as observed in studies similar to this [24,25]. Therefore, these pHs were considered for further studies.

The difference in adsorption capacity of the CV or MB in the adsorbent matrixes at the studied pHs can be related to the type of interaction between the adsorbent/adsorbate. Thus, correlating the results of pH_pzc_, pH and microspecies structures (the distributions of the dye microspecies were obtained using MarvinSketch software 15.4.13) of the dye (CV or MB), a reaction scheme was proposed, taking into account the species CV (Figure S1) and MB (Figure S2) in the adsorbent matrixes studied. Figures S1 and S2 shows that the Cel being added in the dye solution at different pHs can increase the amount of protons in the medium, so to retain charge compensation, a negatively charged biopolymer is consequently generated. Therefore, adsorption of the CV or MB on Cel, at pH 2.0 and 3.0 can be favored by electrostatic interactions, but the competition between the protons and the dye by the surface of the biopolymer makes the adsorption process difficult, since the protons are smaller and interact more easily with the matrix. At pH 4.0 to 6.0, the adsorption increases because it decreases the degree of competition between protons and the dye in the matrix due to the appearance of hydrogen bonds in the adsorption process. At pH 7.0, adsorption is favored strictly by hydrogen bonds and at basic pH by electrostatic interactions and/or hydrogen bonds. On the other hand, as the carboxyl group has a pKa of 3.0 [9], at pH < 3.0, the higher tendency of this group to be protonated is found to hinder the dye adsorption process (CV or MB) in the PhCel matrix at pH 2.0 and 3.0, since there is practically no electrostatic interaction between the dye and the adsorbent matrix. At pH 4.0 to 10.0, the adsorption capacity increases, being favored by electrostatic interactions between the matrix and the dye due to the difficulty of protonation of the carboxylate group at pH > 3.0. The decrease in adsorption capacity of the dye (CV or MB) in the adsorbent matrix may be related to the increase of hydroxyl groups in the medium, generating additional competition between the matrix and these groups by the adsorbate. At extremely basic pH (pH 11.0 and 12.0), dye precipitation may occur due to a large number of hydroxyl groups in the medium, generating a new dye structure [26]. Therefore, for pure cellulose, adsorption of the dye (CV or MB) can be favored by electrostatic interaction and/or hydrogen bonding and in PhCel is favored strictly by electrostatic interactions [27,28].

In addition, it is important to note that the pHpzc of Cel and PhCel is 7.30 and 5.30, respectively. At Ph > pHpzc, the surface of the adsorbent releases protons into the medium at pH < pHpzc, retains protons in the medium. Considering the best pH for the CV adsorption process in Cel is pH 7.0 < pHpzc, and in PhCel is pH 6.0 > pHpzc and adsorption of MB in Cel and PhCel is pH 8.0 > pHpzc, the process is favored exclusively, except in the use of the Cel matrix, due to electrostatic interactions between the charged matrix negatively (pH > pHpzc) and the positively charged dye (CV and MB). At pH < pHpzc, the electrostatic repulsions can occur between the positively charged matrix and the positively charged dye prejudicing the adsorption process, corroborating with the results discussed in Figures S1 and S2 [17,28]. Figure S3 describes the study of the influence of time on the adsorption of crystal violet dye and methylene blue using the Cel or PhCel surface, shows that for pure cellulose, after 100 min contact with crystal violet dye, the amount adsorbed becomes practically constant, which means that for the studied matrix, this time is sufficient for equilibrium between the adsorbent and the adsorbate to be reached. For the adsorption of methylene blue, the equilibration time was 120 min; for the adsorption of CV using the PhCel matrix, 80 min; and for the adsorption of MB, 100 min. The adsorbent/adsorbate equilibrium was observed to be more favorable to electrostatic interactions, as shown in Figures S1 and S2. The experimental results obtained from the study of contact time were submitted to mathematical treatments using different kinetic models (pseudo-first-order [29], pseudo-second-order [30] and Elovich [31]), in order to examine the mechanism that determines the adsorption process, as shown in Figure S3.

Table 1 shows the parameters obtained for the fit of the different kinetic models for the systems studied in aqueous medium. For the adsorption of CV on Cel or PhCel, it is observed that the data obtained experimentally had the best fit to the pseudo-second-order model, since the adjustment with this model shows the highest value of the correlation coefficient (*R*^2^ ≥ 0.9838) compared to the other models, the adsorption of MB in Cel or PhCel, it is observed that the data obtained experimentally had the best fit for the pseudo-first-order model, since the adjustment with this model shows the highest value of the correlation coefficient (*R*^2^ ≥ 0.9845). Also, analysis shows that the experimentally obtained value of the amount adsorbed per gram of adsorbent, *q_e_*_,*exp*_, when compared to the values calculated by the equations of the kinetic models, *q_e,cal_*, indicates that for pseudo-first-order there is a small discrepancy observing adsorption of CV or MB using adsorbent matrices. Thus, the pseudo-first-order model, indicating that diffusion through a boundary precedes the adsorption, is most appropriate to describe the kinetic behavior of the adsorption systems discussed, as has been observed in systems similar to these [32]. Figures S4 and S5 show the adsorption isotherms obtained experimentally, where we observe the temperature does not significantly influence the adsorption of the dye CV (Figure S4) and/or MB (Figure S5) on adsorbent matrices (Cel or PhCel) at temperatures of 298, 308 and 318 K, as observed in similar systems to these [6,7]. The experimental data were adjusted with different isothermal models (Langmuir [33], Freundlich [34] and Temkin [35]), as shown in the Figures S4 and S5. Although the empirical models do not reflect on the issues related to the adsorption mechanism, they provide information on the adsorption capacity of an adsorbent.

Table 2 shows the values of the parameters of the isotherm models for dye adsorption (CV or MB) in aqueous medium. We can observe that the values of the correlation coefficients (*R*^2^), calculated from the nonlinear adjustment to the experimental data by the models, present higher values for the Langmuir model, except for the adsorption isotherm of the CV on PhCel at a temperature of 298 and 308 K, the best fit was the model proposed by Temkin and 318 K, the best fit was the model proposed by Freundlich, as may be observed in the non-linear adjustments shown in Figure S4 (CV) and Figure S5 (MB). For Langmuir model, analyzing the amount of amount adsorbed per gram of adsorbent obtained experimentally, *q_e,exp_*, when compared with the values calculated by the equations of the other models, *q_max_*, indicates that there is a small discrepancy between the adsorption systems studied. Thus, in general, the experimental data have a better fit to the isotherm model proposed by Langmuir, being the Temkin model closer to the Langmuir model, which describes the adsorption process occurring in a monolayer. For all temperatures in the reaction media considered, *R_L_* values are within the range that considers favorable adsorption (0 < *R_L_* < 1). Although the results of the Freundlich model show relatively low correlation coefficient values for some temperatures in the reaction media studied, this corroborates the spontaneity of the adsorption process, since the values of *n_f_* are greater than 1. 

The maximum amount of adsorption of the CV adsorbed on Cel and PhCel was 27.19 and 43.24 mg g^−1^, respectively, and adsorption of MB on Cel and PhCel was 14.37 and 56.99 mg g^−1^, respectively.

## 3. Materials and Methods

### 3.1. Materials

Microcrystalline cellulose (Fagron, São Paulo, Brazil), phthalic anhydride (Isofar, Duque de Caxias, Rio de Janeiro, Brazil), hydrochloric acid (Synth, São Paulo, Brazil), sodium hydroxide (Synth, São Paulo, Brazil), potassium nitrate (Química Moderna Ind., Barueri, Brazil), crystal violet dye (Vetec, CI 42555, Duque de Caxias, Rio de Janeiro, Brazil), methylene blue dye (Proquimios, Bangu, Rio de Janeiro, Brazil), deionized water, all of analytical grade, were used without further purification.

### 3.2. Modification of Cellulose

Carboxylic acid group-containing cellulose was obtained by reaction of pure cellulose (Cel) with phthalic anhydride (Ph), this reaction was adapted from the method described by Melo et al. [20]. The cellulose was reacted with phthalic anhydride (ratio of 1:10 m/m) with stirring (150 rpm) at the melt temperature (405 ± 5 K) of the anhydride for 1 h in the absence of solvent, followed by washing with acetone and deionized water to neutral pH to remove excess reagent and undesirable products. Subsequently, the solid was separated by centrifugation and oven dried for 12 h at 333 K. The final material was termed PhCel.

### 3.3. Determination of Carboxyl Groups Incorporated in Cellulose 

The carboxyl groups incorporated into the cellulose were determined by reverse titration. For this, 0.1 g of PhCel was treated with 100.0 mL of 0.01 mol L^−1^ *NaOH* solution for 1 h under constant magnetic stirring. The solid was separated by centrifugation and three aliquots of 20.0 mL of each of the obtained solutions were titrated with 0.01 mol L^−1^ *HCl* solution [36,37,38]. The concentration of carboxylic groups was calculated using Equation (2):

(2) $$C_{COOH}=\frac{\left(C_{NaOH}V_{NaOH})-(5C_{HCl}V_{HCl}\right)}{m}$$

where *C_NaOH_* and *C_HCl_* are the concetration of the initial hydroxide and hydrochloric acid (mol L^−1^), *V_NaOH_* and *V_HCl_* are the volumes of the initial hydroxide and hydrochloric acid (L) used in the titration of unreacted excess with the base and *m* (g) is the mass of the chemically modified material. 

### 3.4. Zero Point of Charge (pH_pzc_)

The zero point of charge of the matrixes was determined using the solids addition method [2,17]. A 20.0 mL volume of 0.1 mol L^−1^ solution of *KNO*_3_ was added to a series of beakers. The pH of the solution in each vessel was adjusted with *HCl* (0.1 mol L^−1^) and/or *NaOH* (0.1 mol L^−1^) to pH 2.0 to 12.0 and the initial pH of each solution, pH_i_, was measured. To 20.0 mL of each *KNO_3_* solution was added 20.0 mg of the PhCel or Cel matrix. The suspension was stirred (140 rpm) for 24 h at room temperature. After that time, the final pH value of the solution, pH_f_, was measured. The difference between the initial and final pH was calculated: ΔpH = pH_i_ − pH_f_, and the ΔpH plotted as a function of pH_i_. The pH_i_ value with ΔpH equal to zero is called the zero point of charge, pH_pzc_, of the material.

### 3.5. Characterization

The elemental CHN analyses of the samples were recorded using a (Perkin Elmer, Waltham, MA, USA) 2400 Series II instrument. The FTIR spectra were recorded on a MB series FTIR spectrophotometer (Bomem, Ville de Québec, QC, Canada), in transmittance mode with the use of KBr pellets. Measurements were taken in the wavenumber range of 400 cm^−1^ to 4000 cm^−1^, with 32 scans and 4 cm^−1^ resolution. XRD measurements were carried out with a XRD600A diffractometer (Shimadzu, Kyoto, Japan) equipped with Cu-target tube at wavelength λ = 0.1540 nm. Diffractograms were collected at 2θ ranging from 5° to 50° at a rate of 5° min^−1^. Thermogravimetric analyses (TG/DTG) were recorded using a Q600 V20.9 Build TA instrument (TA instrument, New Castle, DE, USA) in the temperature range of 25–1000 °C at a rate of 10 °C min^−1^ under an argon flow rate of 100 mL min^−1^. Absorbance readings were performed in triplicate on an Cary 60 UV-Vis spectrophotometer (Agilent Technologies, Santa Clara, CA, USA), at wavelength with maximum absorption at 581 nm for crystal violet (CV) and 665 nm for methylene blue (MB) colorant and the reported mean values.

### 3.6. Dye Adsorption

#### 3.6.1. Effect of pH

The initial pH variation of the dye solution was studied. For this study, a solution of concentration 100.0 mg L^−1^ of the dye (CV or MB) in the pH range of 2.0 to 10.0 was used. A pH below 2.0 causes the dye solution to change color. A pH above 10.0 causes precipitation to form in the solution. Then, 20.0 mL of dye solution, with pH in the above range, was contacted with 20.0 mg of the matrixes (Cel or PhCel) under stirring (140 rpm) at 298 K in the best time for the adsorption process. After this time, the mixture was centrifuged, aliquots withdrawn from the supernatant solution and the amount of dye remaining determined by absorbance readings. The amount of dye retained in the adsorbent, *q_e_* (mg g^−1^), at each pH, was calculated using Equation (3). The results showed the optimum pHs for adsorbent matrixes in adsorption processes [25,39,40]:

(3) $$q_{e}=\frac{(C_{i}-C_{e})V}{m}$$

where *C_i_* is the initial concentration of the dye (mg L^−1^), *C_e_* is the equilibrium concentration of the dye (mg L^−1^), *m* is the mass of the adsorbent (g) and *V* is the volume of the dye solution (L).

#### 3.6.2. Effect of Time

For the contact time study performed at the best adsorption pH of the biopolymers, 20.0 mL of 100.0 mg L^−1^ (CV or MB) dye solution was placed in contact with 20.0 mg of the Cel or PhCel matrix. This system was kept under stirring (140 rpm) at a temperature of 298 K for different times in the range of 0 to 240 min. the mixtures were centrifuged, aliquots withdrawn from the supernatant solution and the amount of remaining dye determined by absorbance readings. The amount of dye retained, *q_e_* (mg g^−1^) in the adsorbent, for each time, was calculated using Equation (3). The results showed the times required to achieve the maximum adsorption of the adsorbents used [32,41,42]. The experimental data were studied with pseudo-first-order [29], pseudo-second-order [30] and Elovich [31] equations to verify the kinect of the adsorption process. For the nonlinear adjustment of the time isotherm to the pseudo-first-order model the Equation (4) is used in the non-linear form developed by Lagergren [29]:

(4) $$q_{t}=q_{e,cal}\left[1-\exp(-k_{1}t)\right]$$

where *q_e,exp ou cal_* (mg g^−1^) is the amount adsorbed per gram of adsorbent, *q_t_* (mg g^−1^) is the amount adsorbed per gram of adsorbent in the time *t* (min) and *k*_1_ (min^−1^) is the pseudo-first-order adsorption rate constant [29]. 

The nonlinear adjustment of the experimental data using the pseudo-second-order model, developed by Ho and McKay [30], can be performed using Equation (5) and the initial adsorption rate, *h* (mg g^−1^ min^−1^), when *t* = 0 can be obtained by Equation (6):

(5) $$q_{t}=\frac{k_{2}q_{e,cal}{}^{2}t}{1+q_{e,cal}k_{2}t}$$

(6) $$h=k_{2}{q_{e,cal}}^{2}$$

where *k*_2_ (g mg^−1^ min^−1^) is the pseudo-second-order rate constant [30]. The Elovich model [31], represented by Equation (7), is suitable for systems whose adsorption surfaces are heterogeneous and show different activation energies in the chemisorption process:

(7) $$q_{t}=\frac{1}{\beta }\ln(\alpha \beta t)$$

where *β* (g mg^−1^) is the adsorption constant related to the degree of coverage of the adsorbent surface and the activation energy of the chemisorption process, *α* (mg g^−1^ min^−1^) is the initial adsorption velocity constant and *q_t_* (mg g^−1^) is the amount adsorbed at time *t* (min) [31].

#### 3.6.3. Effect of Dye Concentration and Temperature

In the study of the influence of the concentration on the adsorption process, 20.0 mg of the matrices (Cel or PhCel) was placed in contact with 20.0 mL of dye solution (CV or MB) at different concentrations in the range of 0 to 100.0 mg L^−1^, at the optimum pH and for the optimum times for adsorption. The system was kept under stirring (140 rpm) at different temperatures (298, 308 and 318 K). After the optimum times, the mixtures were centrifuged, aliquots removed from the supernatant solution and the absorbances read. The amount of dye retained in the adsorbent, *q_e_* (mg g^−1^), at each concentration was calculated using Equation (3). The adjustment of the experimental data of the concentration isotherms was performed using the isothermal nonlinear models of Langmuir [33], Freundlich [34] and Temkin [35].

For the adjustment of the experimental concentration isotherm to the model proposed by Langmuir [33], Equation (8) is used in the nonlinear form. The parameters of the Langmuir equation can be expressed in terms of a dimensionless separation factor, *R_L_*, defined by Equation (9), which can then evaluate the isotherm shape and the spontaneity of the adsorption process: *R_L_* 〉  1 (unfavorable adsorption process), *R_L_* = 1 (linear adsorption process), 0  〈  *R_L_*  〈  1 (favorable adsorption process) and *R_L_* = 0 (irreversible adsorption process) [33]:

(8) $$q_{e}=\frac{K_{L}q_{\max}C_{e}}{1+K_{L}C_{e}}$$

(9) $$R_{L}=\frac{1}{1+K_{L}C_{e}}$$

where *q_e_* (mg g^−1^) is the equilibrium adsorption capacity, *C_e_* (mg L^−1^) is the equilibrium concentration of the dye, *q_max_* (mg g^−1^) is the maximum amount of theoretical adsorption at equilibrium and *K_L_* (L mg^−1^) is Langmuir constant [33]. 

The adjustment of the experimental data using the Freundlich model [34] can be evaluated using Equation (10) in the nonlinear form:

(10) $$q_{e}=K_{f}C_{e}{}^{\left(1/n_{f}\right)}$$

where *q_e_* and *C_e_* have the same meaning as in the Langmuir equation, *K_f_* (L g^−1^) is a constant related to the adsorption capacity and *n_f_* is a constant related to the adsorption intensity and the spontaneity of the adsorption, when this value is greater than 1. The experimental data can be adjusted to the Temkin model [35] using Equation (11) in nonlinear form:

(11) $$q_{e}=\frac{RT}{b_{T}}\ln(A_{T}C_{e})$$

where *q_e_* and *C_e_* have the same meaning as in the Langmuir equation, *T* (K) is the temperature, *R* (8.314 J mol^−1^ K^−1^) is the gas constant, *A_T_* (L mg^−1^) and *b_T_* (J mol^−1^) are the isotherm constant and Temkin constant, respectively [35]. 

## 4. Conclusions

In the search for matrixes that could be used in the removal of cationic dyes in aqueous solution, the reaction to obtain the phthalic anhydride-modified cellulose matrix (PhCel) was confirmed by product characterization (elemental analysis, FTIR, XRD, TG/DTG and determination of carboxylic acid groups). In the adsorption tests carried out using 20.0 mg of the adsorbent matrix and 20.0 mL of the dye solution (CV or MB), the PhCel matrix was efficient in the adsorption of CV or MB in aqueous medium when compared to the pure biopolymer (Cel).

In the time study, the data obtained experimentally had the best fit to a pseudo-first-order model. The concentration isotherms obtained had the best fit to the model proposed by Langmuir, at temperatures of 298, 308 and 318 K, except for the adsorption isotherm of the CV on PhCel at a temperature of 298 and 308 K, where the best fit was to the model proposed by Temkin and 318 K, where the best fit was the model proposed by Freundlich. The adsorption capacities of CV on Cel (pH 7.0 and 100 min) and PhCel (pH 6.0 and 80 min) were 27.19 and 43.24 mg g^−1^, respectively, and the adsorptions of MB on Cel (pH 8.0 and 120 min) and PhCel (pH 8.0 and 100 min) were 14.37 and 56.99 mg g^−1^, respectively. 

The adsorption of the CV or MB dye on PhCel can be favored strictly by electrostatic interactions in comparison with Cel, in which the adsorption can be favored by electrostatic interactions and/or hydrogen bonds. Therefore, Cel and PhCel can be used in the removal of CV or MB dyes in the environment, since these materials have good adsorption capacity. 

## Figures and Tables

**Table 1:** Modeled kinetic parameters for the adsorption of CV and MB by Cel or PhCel.

Adsorbent	pH	Dye	*q_e,exp_* (mg g^−1^)	Pseudo-First-Order Model			Pseudo-Second-Order Model				Elovich Model		
				*k*_1_ (min^−1^)	*q_e,cal_* (mg g^−1^)	*R*^2^	*k*_2_ (g mg^−1^ min^−1^)	*q_e,cal_* (mg g^−1^)	*h* (mg g^−1^ min^−1^)	*R*^2^	*α* (mg g^−1^ min^−1^)	*β* (g mg^−1^)	*R*^2^
Cel	7.0	CV	24.7470	0.0328	24.5225	0.9815	0.0014	28.7321	1.1557	0.9879	3.3022	0.1788	0.9829
	8.0	MB	9.6460	0.0201	10.2337	0.9892	0.0016	12.8901	0.2658	0.9861	0.5198	0.3359	0.9818
PhCel	6.0	CV	34.8730	0.0460	34.1093	0.9795	0.0019	37.9294	2.7334	0.9838	18.6190	0.1769	0.9741
	8.0	MB	41.7840	0.0262	43.5332	0.9845	0.0005	53.2477	1.4177	0.9661	2.8421	0.0823	0.9512

**Table 2:** Isotherm model parameters for the adsorption of CV and MB on Cel or PhCel.

Adsorbent	pH	Dye	Temperature (K)	Langmuir				Freundlich			Temkin		
				*q_max_* (mg g^−1^)	*K_L_* (L mg^−1^)	*R_L_*	*R*^2^	*n_f_*	*K_f_* (L g^−1^)	*R*^2^	*A_T_* (L mg^−1^)	*b_T_* (J mol^−1^)	*R*^2^
Cel	7.0	CV	298	27.1985	0.1403	0.1336	0.9844	3.5521	7.8022	0.9648	1.9197	110.1706	0.9843
			308	26.6632	0.1354	0.1696	0.9700	3.7394	8.0199	0.9541	2.6169	151.0012	0.9690
			318	27.0856	0.1166	0.1538	0.9784	3.4158	7.2187	0.9637	1.7727	166.9936	0.9783
Cel	8.0	MB	298	12.0854	0.0671	0.2193	0.9909	2.7744	2.1758	0.9615	0.6367	216.2711	0.9863
			308	14.3716	0.0549	0.2446	0.9886	2.4717	2.0835	0.9558	0.5589	228.1585	0.9811
			318	14.1790	0.0731	0.2240	0.9910	2.7743	2.6760	0.9604	0.7447	278.4868	0.9857
PhCel	6.0	CV	298	37.5097	0.2686	0.1067	0.9682	4.0826	13.6859	0.9666	7.6652	96.5475	0.9790
			308	37.1611	0.2677	0.0881	0.9637	4.0281	13.4287	0.9820	9.8824	126.9822	0.9840
			318	43.2401	0.0696	0.2596	0.9805	2.4657	6.8735	0.9849	1.1053	103.9071	0.9699
PhCel	8.0	MB	298	52.2047	0.1590	0.1885	0.9256	3.5962	16.0065	0.8550	1.7601	53.5470	0.8930
			308	56.9999	0.0847	0.2935	0.9448	2.4163	9.4411	0.8712	0.6308	51.7234	0.9418
			318	55.0383	0.1428	0.3313	0.9567	2.8477	12.7742	0.8709	1.2046	69.4659	0.9360

## Supplementary Materials

The following are available online, Figure S1: (i) Distribution of CV microspecies under different pHs. Scheme of adsorption process of CV in the (ii) Cel and (iii) PhCel under different pHs, Figure S2: (i) Distribution of MB microspecies under different pHs. Scheme of adsorption process of MB in the (ii) Cel and (iii) PhCel under different pHs, Figure S3: Effect of contact time on the adsorption of CV (a and b) and MB (c and d) onto Cel (- ■ -) or PhCel (- ● -) and the nonlinear adjustments of kinetic models, Figure S4: Effect of CV concentration on the adsorption process in the Cel (a–c) or PhCel (d–f) in different temperatures and the nonlinear adjustments of Isotherm models, Figure S5: Effect of MB concentration on the adsorption process in the Cel (a–c) or PhCel (d–f) in different temperatures and the nonlinear adjustments of Isotherm models.
