# Supplementary material for: Potential of Cellulose Functionalized with Carboxylic Acid as Biosorbent for the Removal of Cationic Dyes in Aqueous Solution
Source: Molecules. 2018 Mar 23;23(4):743. doi: 10.3390/molecules23040743 (PMC6017135; doi:10.3390/molecules23040743)
Supplement: Supplementary file 1 [file molecules-23-00743-s001.pdf]

Supplementary material

Potential of Cellulose Functionalized with Carboxylic Acid as Biosorbent for the Removal of Cationic Dyes in Aqueous Solution

Lucinaldo dos Santos Silva [1], Jhonatam de Oliveira Carvalho [1], Roosevelt Delano Sousa Bezerra [2], Mateus Soares da Silva [3], Francisco José Lustosa Ferreira[3], Josy Anteveli Osajima [3] and Edson Cavalcanti da Silva Filho [3,*]

[1] Açailândia Campus, Federal Institute of Maranhão, IFMA, Açailândia, MA 65930-000, Brazil; ssdlucinaldo@gmail.com (L.d.S.S.); jhonatam.carvalho@ifma.edu.br (J.d.O.C.)
[2] Teresina-Central Campus, Federal Institute of Piauí, IFPI, Teresina, PI 64000-040, Brazil; rooseveltdsb@ifpi.edu.br
[3] Interdisciplinary Laboratory for Advanced Materials—LIMAV, UFPI, Teresina, PI 64049-550, Brazil; mateusufpi@gmail.com (M.S.d.S.); franciscojufpi@gmail.com (F.J.L.F.); josy_osajima@yahoo.com.br (J.A.O.)
* Correspondence: edsonfilho@ufpi.edu.br; Tel.: +55-86-3221-5710

Contents of the supplementary material:

(i) Distribution of CV microspecies under different pHs. Scheme of adsorption process of CV in the (ii) Cel and (iii) PhCel under different pHs...Figure S1
(i) Distribution of MB microspecies under different pHs. Scheme of adsorption process of MB in the (ii) Cel and (iii) PhCel under different pHs...Figure S2
Effect of contact time on the adsorption of CV (a and b) and MB (c and d) onto Cel (- ■ -) or PhCel (- ● -) and the nonlinear adjustments of kinetic models...Figure S3
Effect of CV concentration on the adsorption process in the Cel (a–c) or PhCel (d–f) in different temperatures and the nonlinear adjustments of Isotherm models....................Figure S4
Effect of MB concentration on the adsorption process in the Cel (a–c) or PhCel (d–f) in different temperatures and the nonlinear adjustments of Isotherm models....................Figure S5

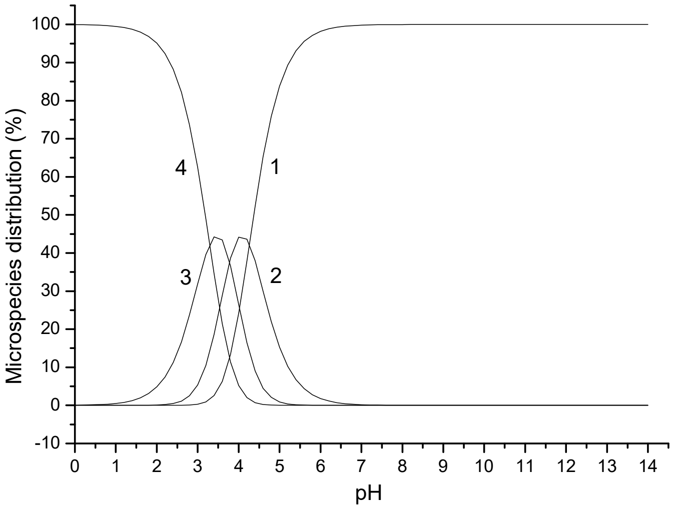
Microspecies distribution (%)
pH
1
2
3
4
100
90
80
70
60
50
40
30
20
10
0
-10
0 1 2 3 4 5 6 7 8 9 10 11 12 13 14

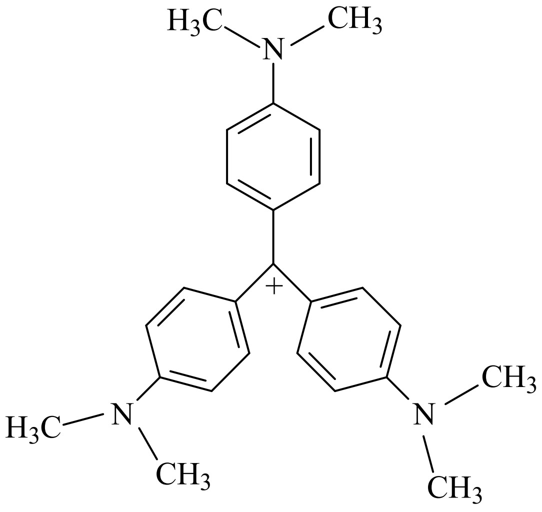

(1)

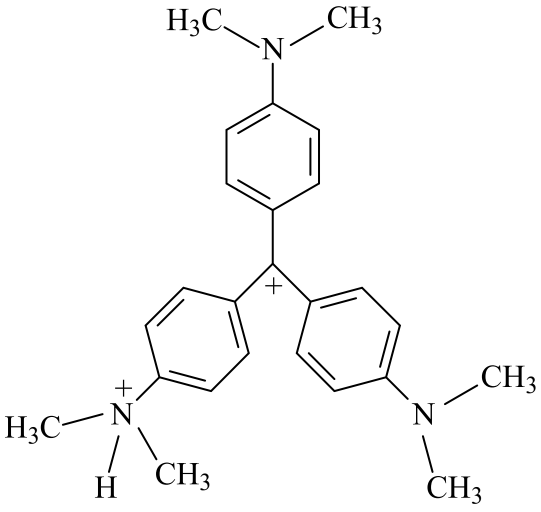

(2)

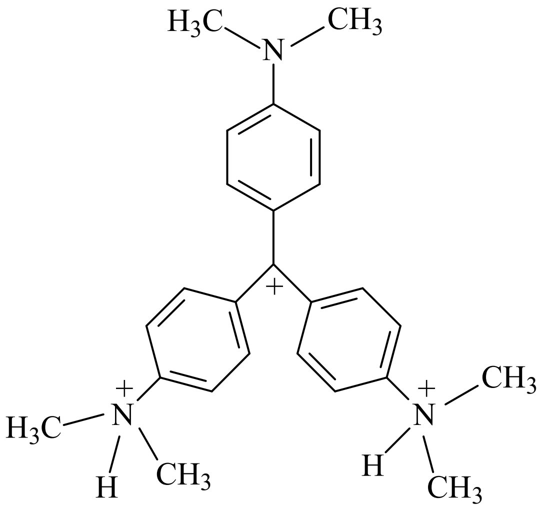

(3)

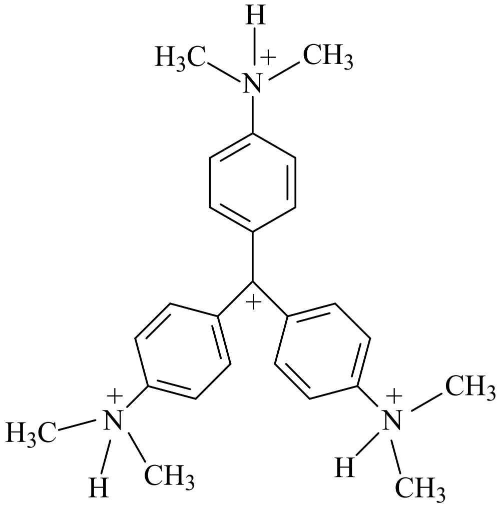

(4)

(i)

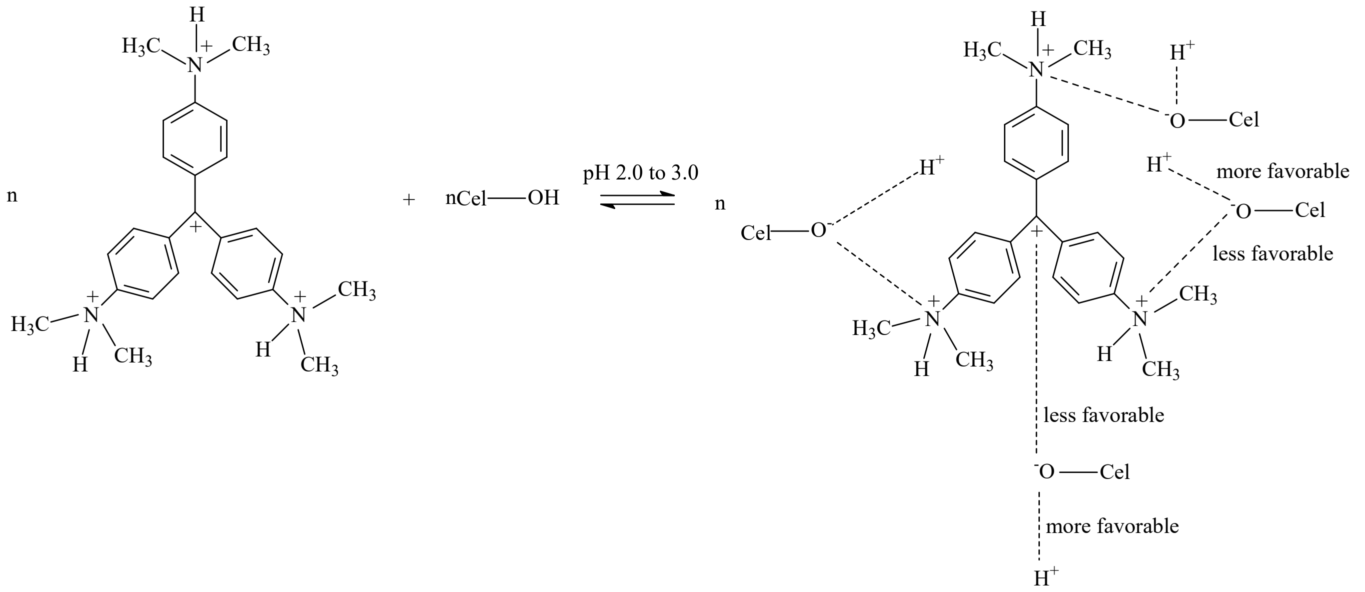
pH 2.0 to 3.0
nCel—OH
more favorable
less favorable
less favorable
more favorable

(1)

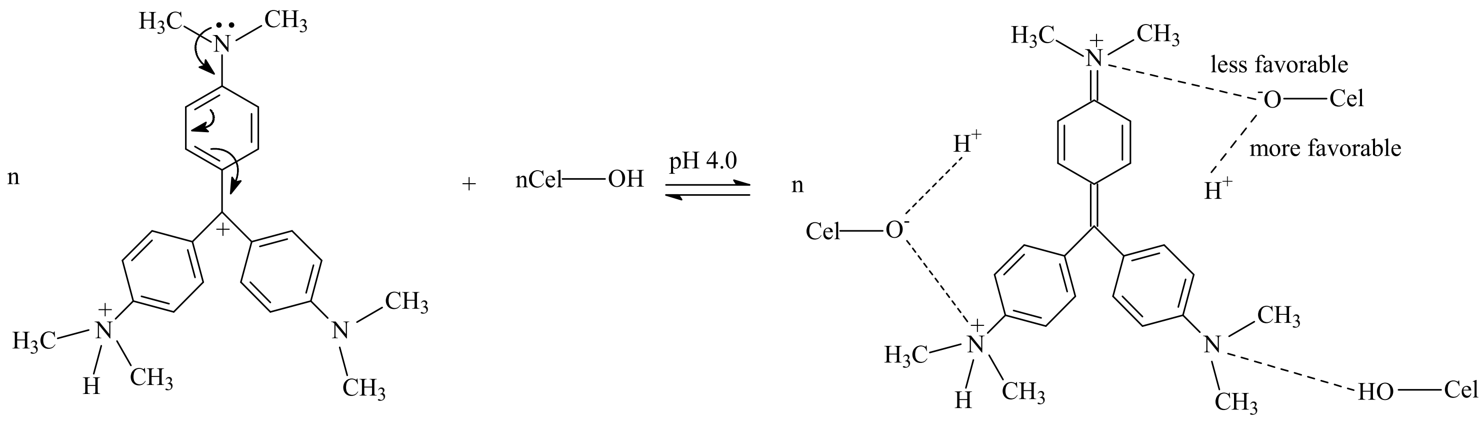
pH 4.0
nCel—OH
less favorable
more favorable

(2)

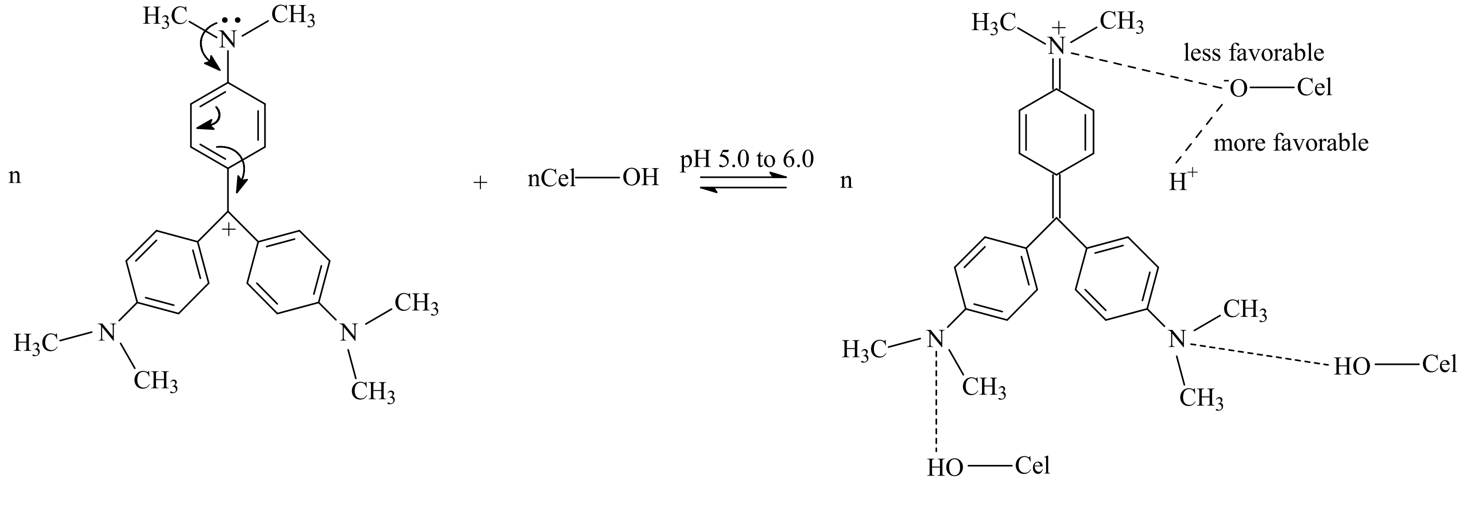
pH 5.0 to 6.0
nCel—OH
less favorable
more favorable

(3)

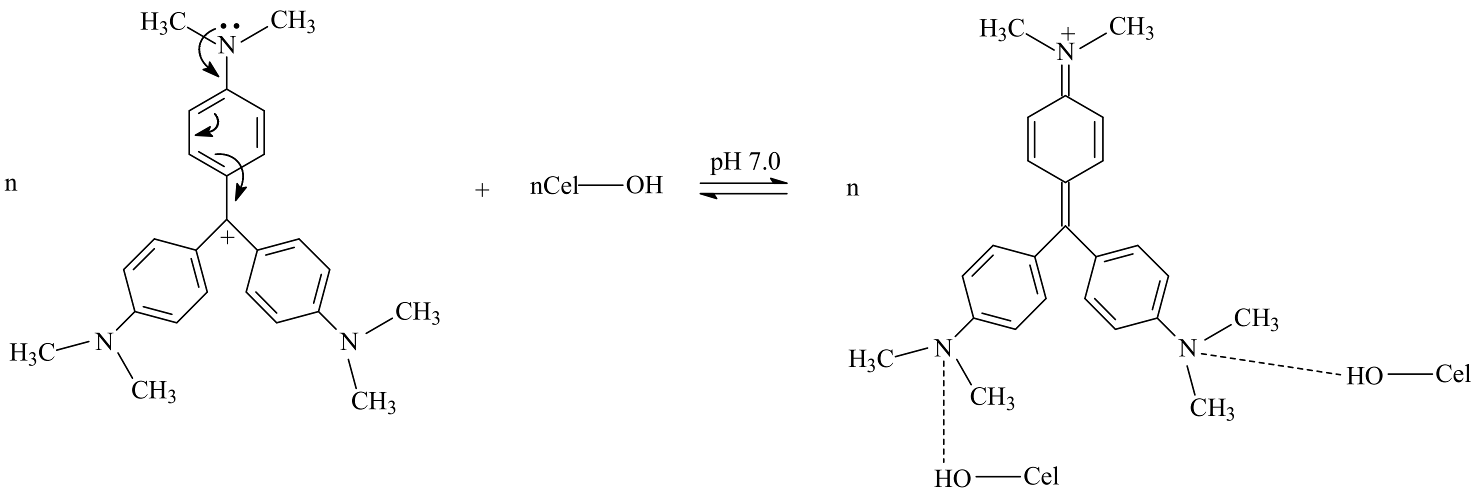
pH 7.0
nCel—OH
HO—Cel
HO—Cel

(4)

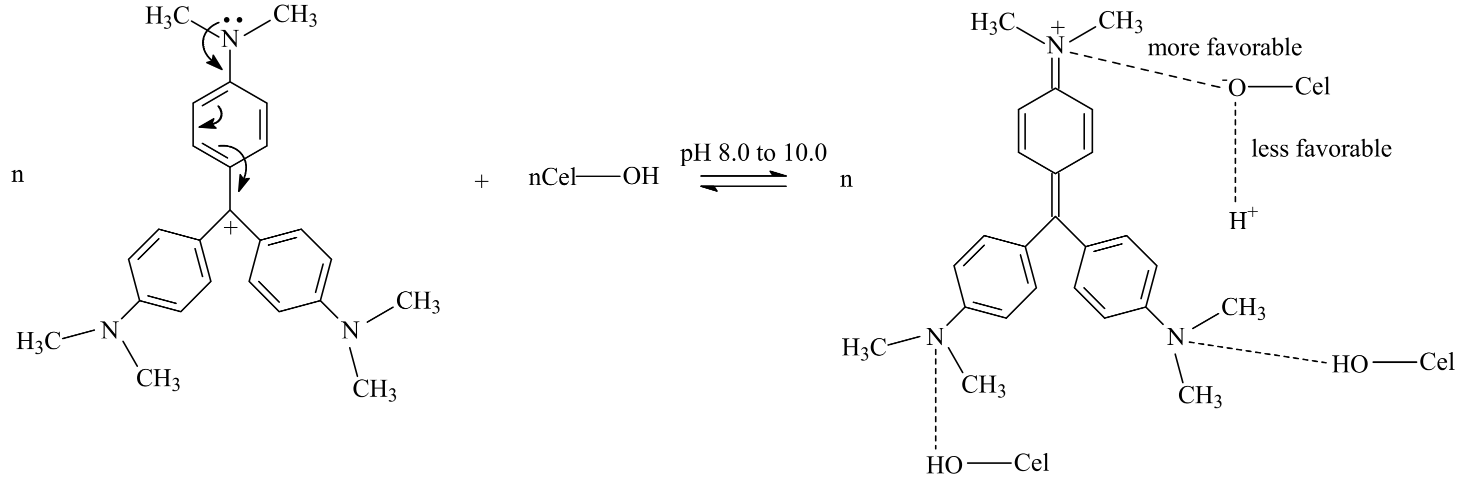
pH 8.0 to 10.0
nCel—OH
more favorable
less favorable
HO—Cel
HO—Cel

(5)

(ii)

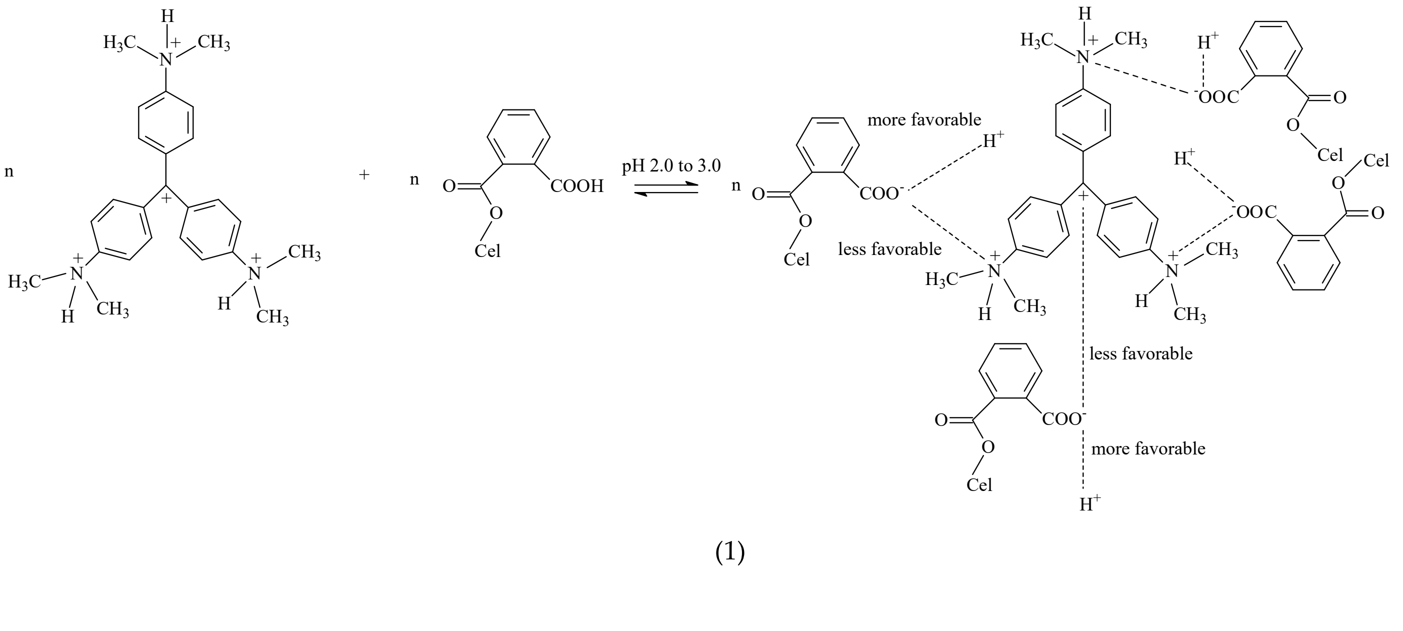
pH 2.0 to 3.0
more favorable
less favorable
less favorable
more favorable

(1)

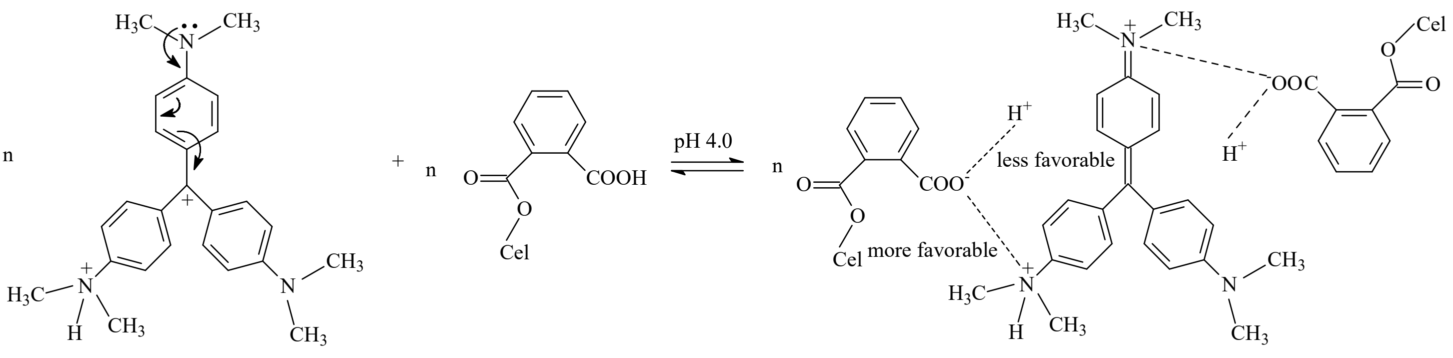

(2)

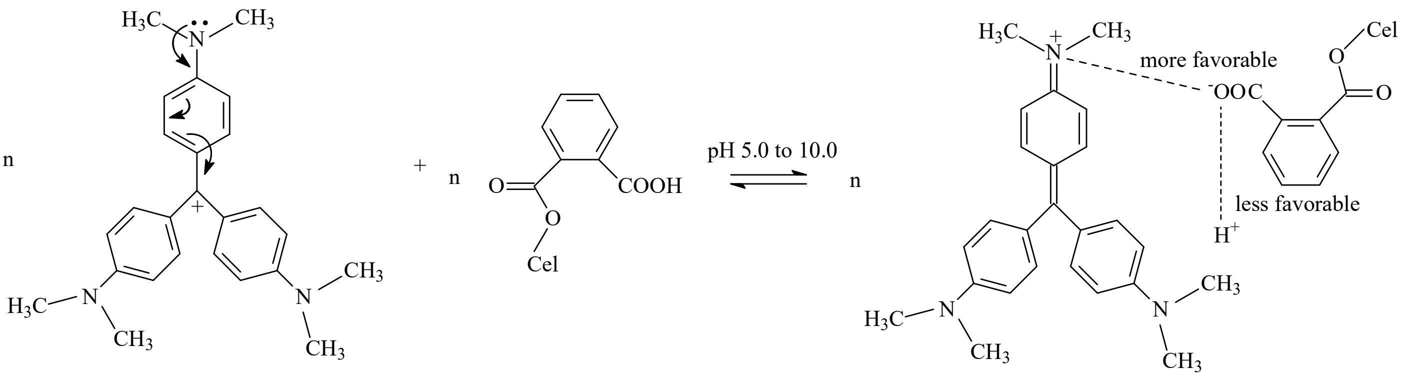

(3)

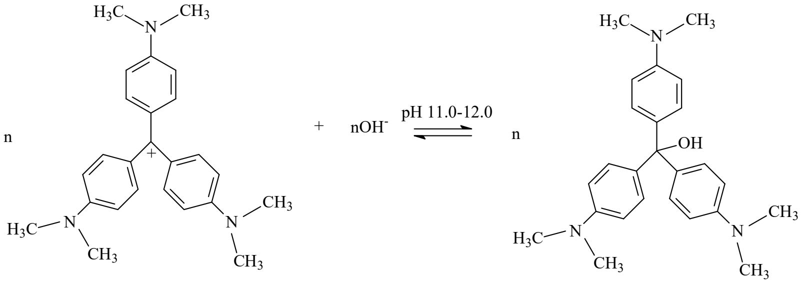

(4)

(iii)

Figure S1. (i) Distribution of CV microspecies under different pHs. Scheme of adsorption process of CV in the (ii) Cel and (iii) PhCel under different pHs.

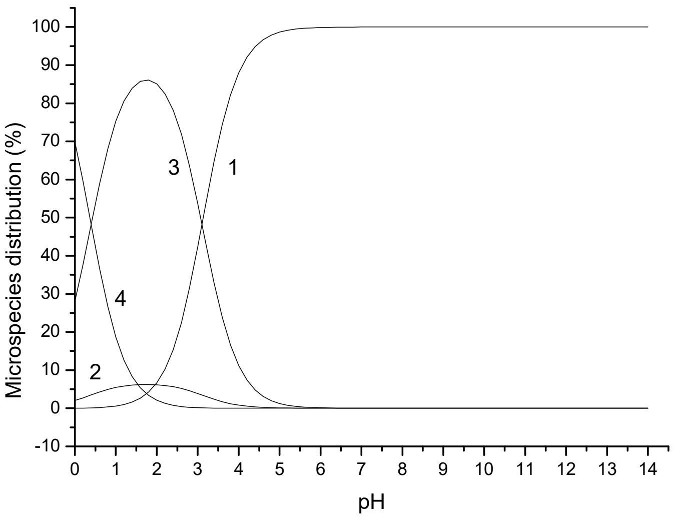
Microspecies distribution (%)
pH
1
2
3
4

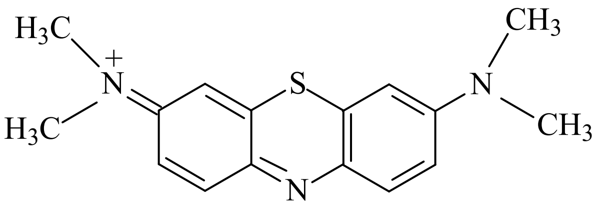

(1)

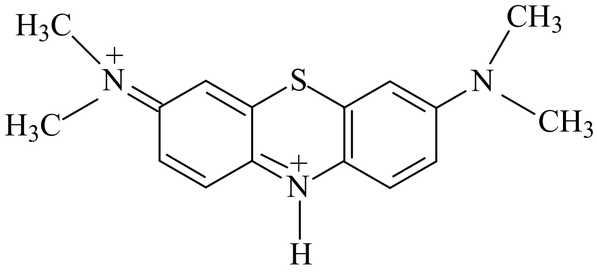

(2)

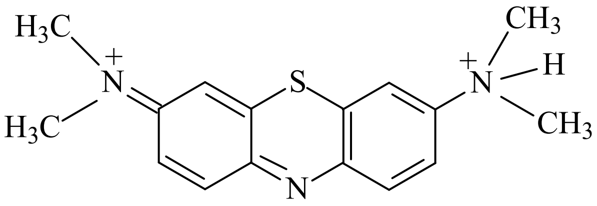

(3)

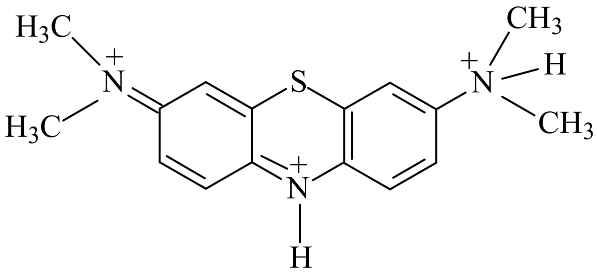

(4)

(i)

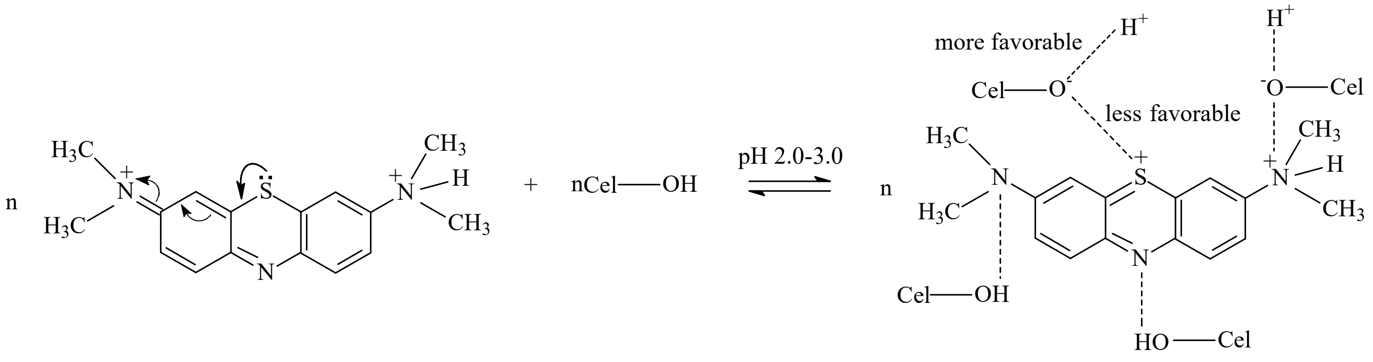
more favorable
less favorable
pH 2.0-3.0
nCel—OH

(1)

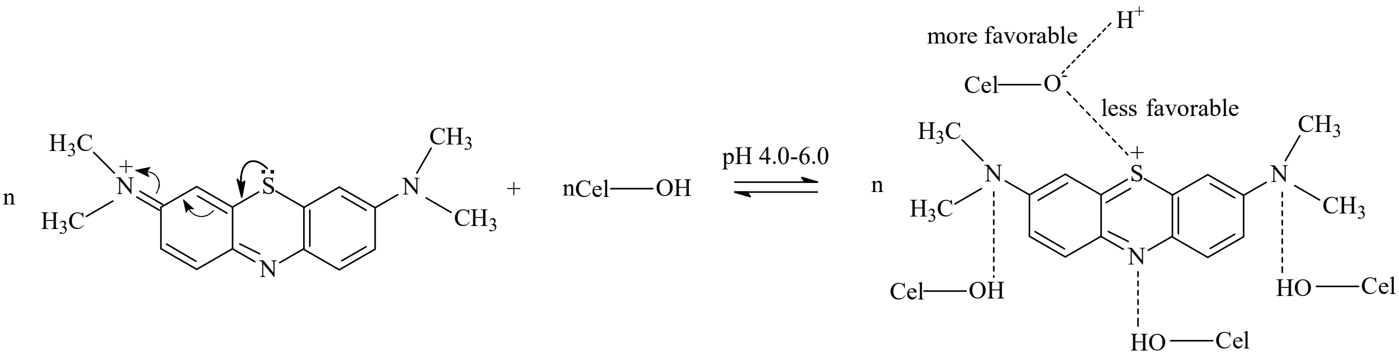
more favorable
less favorable
pH 4.0-6.0

(2)

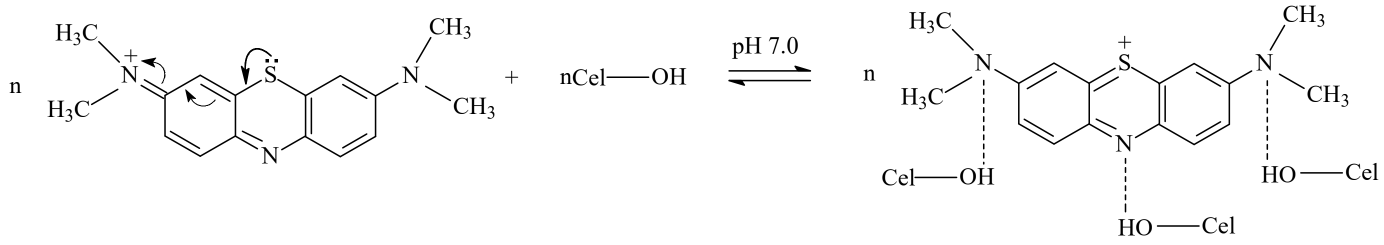
pH 7.0

(3)

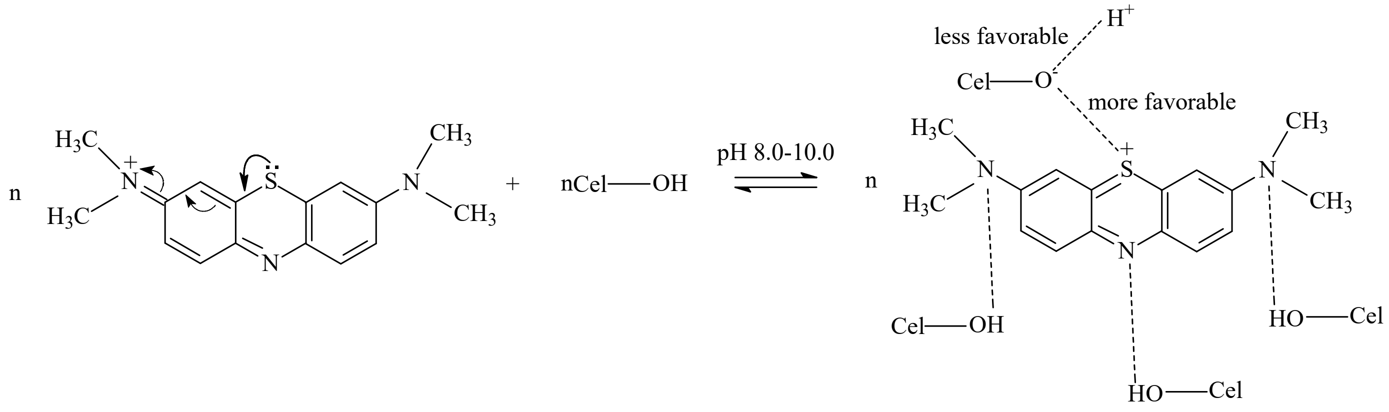
less favorable
more favorable
pH 8.0-10.0

(4)

(ii)

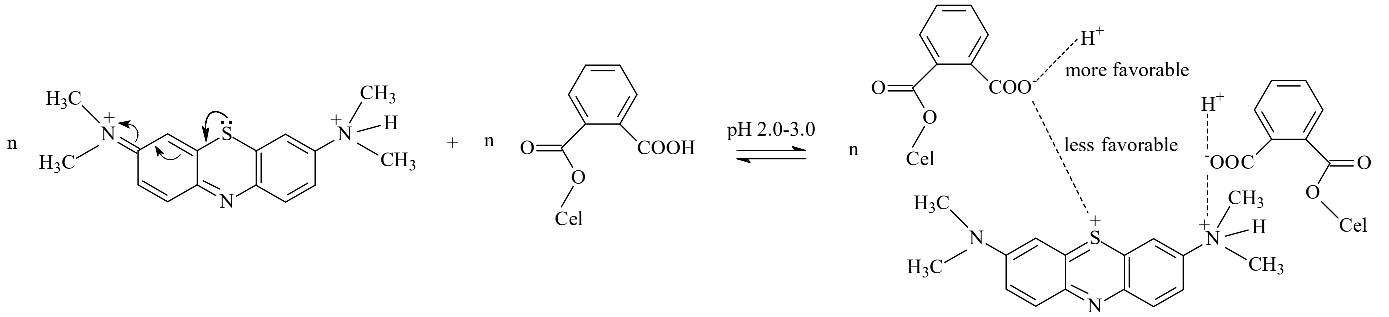
pH 2.0-3.0
more favorable
less favorable

(1)

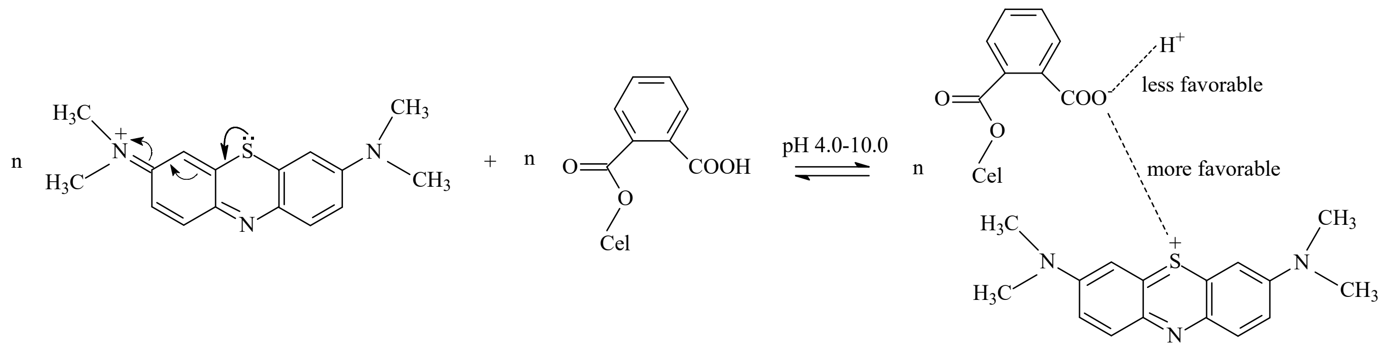

(2)

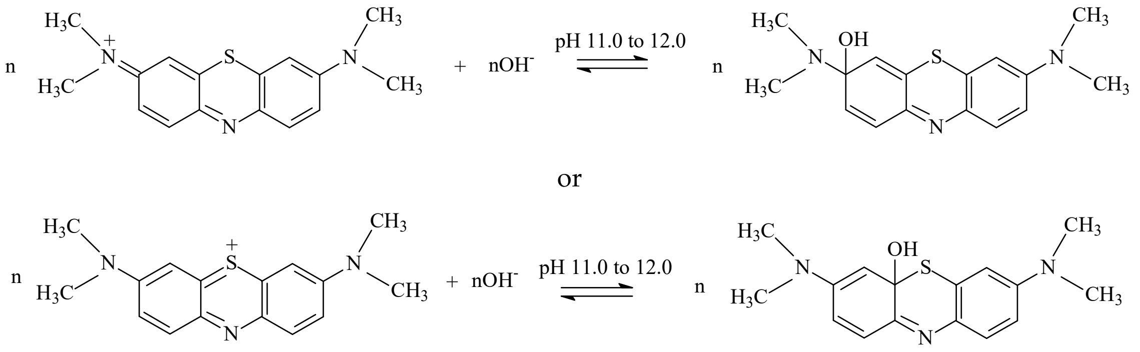

(3)

(iii)

Figure S2. (i) Distribution of MB microspecies under different pHs. Scheme of adsorption process of MB in the (ii) Cel and (iii) PhCel under different pHs.

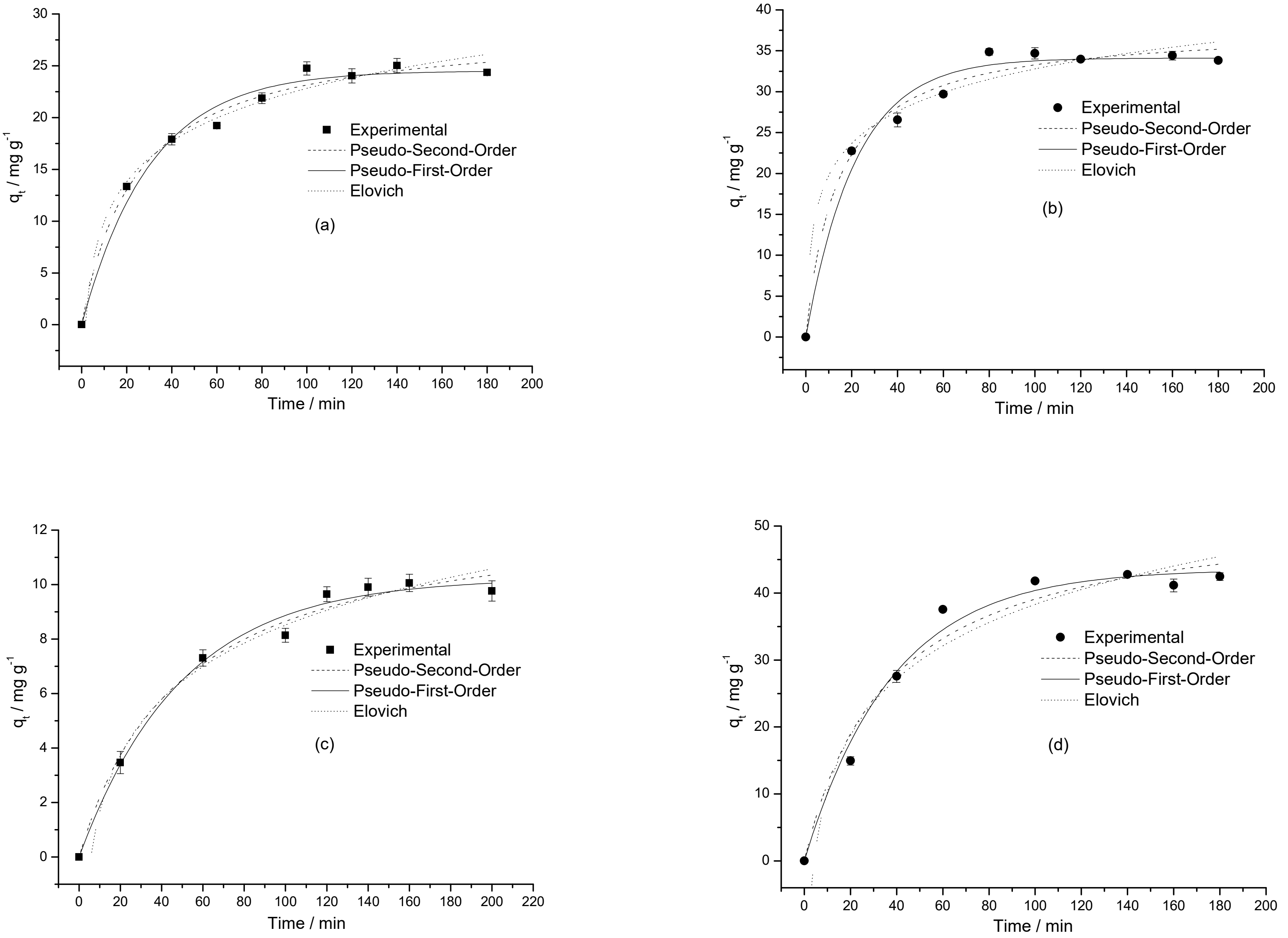

Figure S3. Effect of contact time on the adsorption of CV (a and b) and MB (c and d) onto Cel (- ■ -) or PhCel (- ● -) and the nonlinear adjustments of kinetic models.

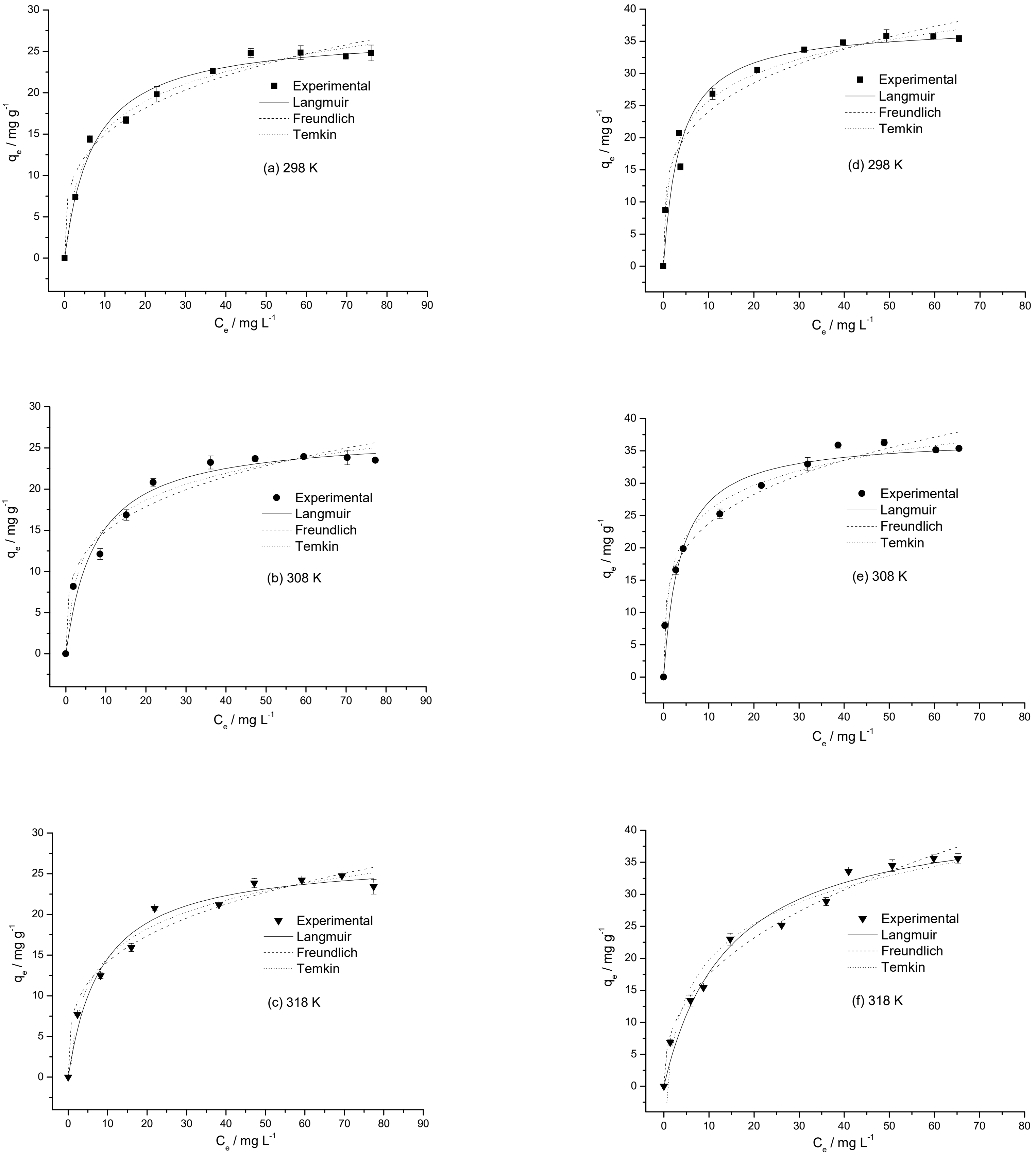
Experimental
Langmuir
Freundlich
Temkin
(a) 298 K
(b) 308 K
(c) 318 K
(d) 298 K
(e) 308 K
(f) 318 K
q_e / mg g^{-1}
C_e / mg L^{-1}

Figure S4. Effect of CV concentration on the adsorption process in the Cel (a–c) or PhCel (d–f) in different temperatures and the nonlinear adjustments of Isotherm models.

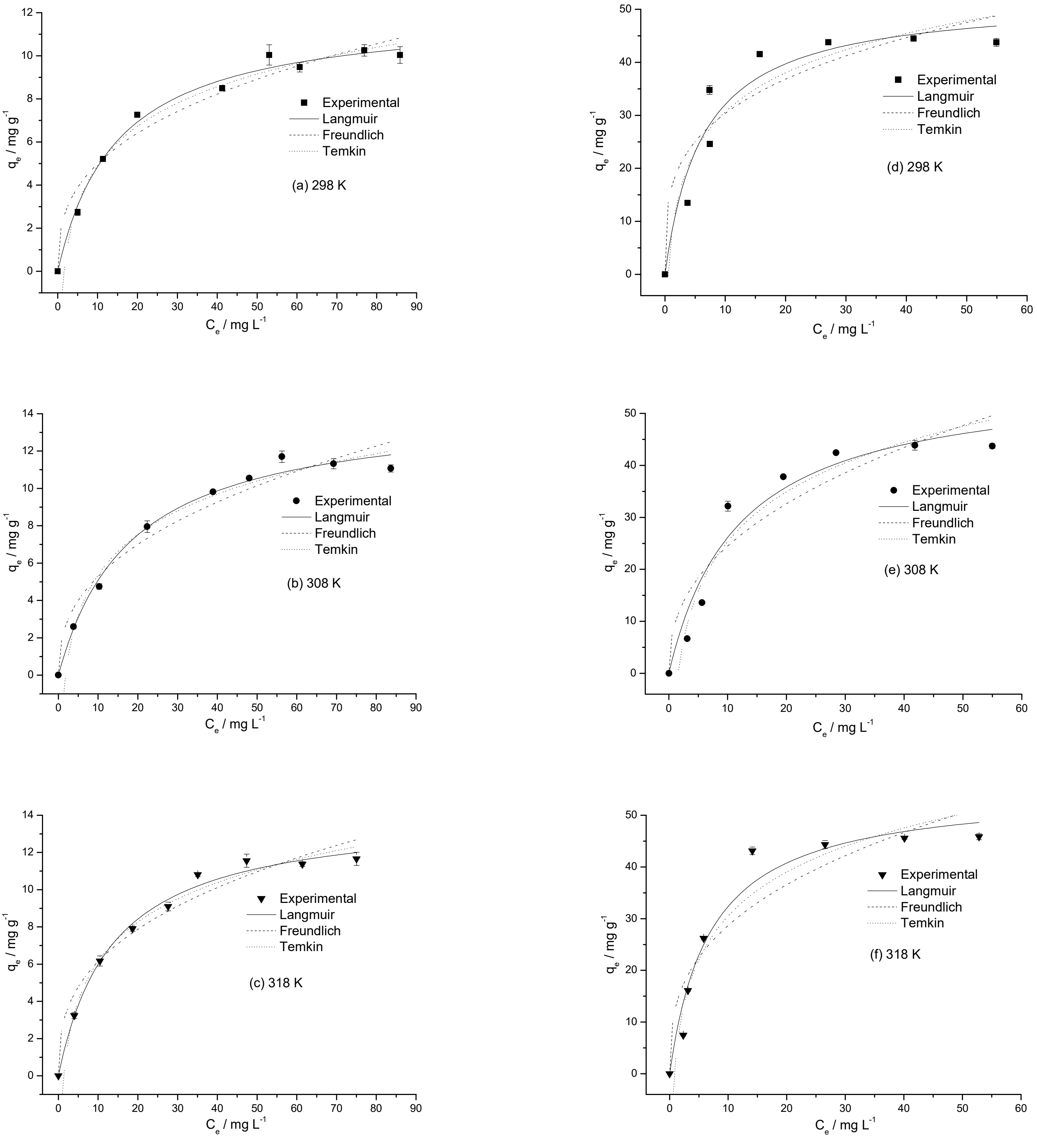

Figure S5. Effect of MB concentration on the adsorption process in the Cel (a–c) or PhCel (d–f) in different temperatures and the nonlinear adjustments of Isotherm models.
